# Midwives’ views on implementation of antiretroviral clinical guidelines for the prevention of HIV in a private hospital

**DOI:** 10.4102/hsag.v31i0.3229

**Published:** 2026-04-30

**Authors:** Tshegofatso E. Chauke, Maurine R. Musie, Fhumulani M. Mulaudzi

**Affiliations:** 1 Department of Nursing Science, Faculty of Health Sciences, University of Pretoria, Pretoria, South Africa

**Keywords:** antiretroviral therapy, HIV, HIV clinical guidelines, maternity wards, midwives, private hospitals, vertical transmission prevention

## Abstract

**Background:**

South Africa has made considerable progress in HIV and AIDS management, particularly in vertical transmission prevention (VTP). The 2023 national HIV guidelines, which includes provision for pregnant and breastfeeding women, mark an important advancement. However, implementation in the private healthcare sector remains uncertain. The effective application of antiretroviral therapy (ART) guidelines in maternity wards within private hospitals is essential to prevent vertical transmission of HIV.

**Aim:**

This study explored midwives’ perspectives on the implementation of ART clinical guidelines for preventing vertical HIV transmission in maternity wards within a private hospital in Gauteng province.

**Setting:**

The study was conducted in a maternity ward of a private hospital in the Tshwane District, Gauteng, South Africa.

**Methods:**

A qualitative, exploratory, descriptive design was used. Thirteen purposively selected midwives participated in in-depth, semi-structured interviews, which were audio-recorded with consent, transcribed verbatim and thematically analysed to develop themes.

**Results:**

Four themes emerged: (1) Lack of standardised practice in implementing VTP guidelines; (2) Ethical and confidentiality challenges; (3) Limited role of midwives in VTP management; and (4) Strategies to enhance implementation and use of ART clinical guidelines in private hospitals.

**Conclusion:**

The findings highlight challenges in implementing ART guidelines in private maternity settings and emphasise the need for stronger integration of national HIV guidelines through training, policy alignment and institutional support to improve maternal and neonatal outcomes.

**Contribution:**

The study presents recommendations for strengthening the role of private hospitals in reducing vertical HIV transmission and supporting HIV and AIDS elimination goals.

## Introduction

Preventing vertical transmission (VTP) of HIV from mother to child is an important public health objective, particularly in areas where HIV prevalence is high. According to the report by the United States Agency for International Development, in the 2023 report, 39.9 million people globally were living with HIV, and 1.3 million people became newly infected with HIV (Swinkels, Nguyen & Gulick 2024). Antiretroviral therapy (ART) is a very successful intervention in reducing HIV transmission from mother-to-child during pregnancy, childbirth and breastfeeding (Xu et al. 2024). As a result, the ART clinical guidelines (National Department of Health 2023a), and guidelines for VTP of communicable infections (National Department of Health 2023b), have been developed to provide healthcare providers, including midwives, with evidence-based protocols to ensure the successful implementation of these preventive guidelines (Herce et al. 2024; World Health Organization 2022). The implementation of clinical guidelines for ART is crucial for ensuring consistent, high-quality care across healthcare settings. Currently in South Africa, the National Department of Health has developed ART clinical guidelines (National Department of Health 2023a), and national guidelines for VTP of communicable infections (National Department of Health 2023b), to standardise practice, improve patient safety and enhance maternal and neonatal outcomes (Aphane et al. 2025). These guidelines emphasise early HIV testing at initial antenatal booking, immediate initiation of ART for women who test positive, routine monitoring of viral load and CD4 count, screening for co-infections such as tuberculosis and continued follow-up throughout pregnancy, labour and the breastfeeding period. It is recommended that testing be repeated on each scheduled visit to antenatal care for those who tested negative. Effective implementation of these guidelines is critical for reducing transmission risk and improving birth outcomes. Upon arrival in the delivery room, however, the adoption and implementation of these guidelines vary significantly across different healthcare institutions, particularly in private hospital maternity wards, where resources, priorities and patient needs may differ from those in public healthcare facilities. The scope of practice of midwives in South Africa is regulated by the South African Nursing Council (2001), which authorises them to provide comprehensive maternal and newborn care, including HIV counselling and testing, initiation of certain treatments aligned with national programmes and ongoing maternal monitoring. In the public healthcare sector, midwives frequently function as primary maternity care providers and are expected to implement national programmes autonomously. In contrast, private hospital maternity care is often obstetrician-led, with midwives functioning more in supportive or monitoring roles. These structural differences may influence how ART and VTP guidelines are interpreted and applied in private maternity settings. The gap noted in the literature is that research has focused on guideline implementation in public healthcare settings and limited attention has been given to maternity wards within private hospitals, where healthcare dynamics, resource allocation and patient demographics may differ. The World Health Organization (2023) has noted that implementation of ART clinical guidelines can differ across sectors, with maternity ward within private hospitals sometimes lacking the same structured programme monitoring systems found in public healthcare facilities. Such variability may lead to inconsistencies in care and potentially affect patient outcomes. Maternity wards within private hospitals may not always adhere to the same structured protocols as public institutions. In many countries, private hospitals play a significant role in delivering healthcare services and often serve diverse populations with varied socioeconomic backgrounds, who may have different needs and access to resources than those in public healthcare settings (Ghasemi et al. 2022). In South Africa, approximately 5% – 14% of births occur in private healthcare facilities, with the majority taking place in the public sector (National Department of Health et al. 2016). Globally, the proportion of births occurring in private facilities varies widely but remains a minority in most low- and middle-income countries (Gage et al. 2025). The differences in institutional priorities, staffing models and resource allocation may contribute to variability in the application and adherence to the ART clinical guidelines potentially leading to suboptimal patient care or even adverse outcomes (Brautsch et al. 2023). As a result, there is a growing need to explore how ART clinical guidelines are implemented in maternity wards within private hospitals, the challenges faced by healthcare providers in adhering to these guidelines and the impact of such adherence on patient outcomes. Midwives, as primary caregivers during pregnancy and childbirth, are in a unique position to influence the outcome of HIV prevention strategies (Carbonell et al. 2024). Their role in implementing the antiretroviral clinical guidelines for VTP is paramount, as they are directly involved in maternal care, counselling and ensuring the administration of ART during antepartum, intrapartum, postpartum and breastfeeding periods (Aba Abraham & Clow 2022) as illustrated in the guidelines for VTP of communicable infections (National Department of Health 2023b). However, the successful implementation of these guidelines is not without challenges and is influenced by multiple factors, including healthcare provider attitudes, institutional policies, training opportunities and resource availability (Mody et al. 2024). According to Mody et al. (2024) and Musie and Mulaudzi (2024), the attitudes, perceptions and views of midwives regarding the antiretroviral clinical guidelines can significantly impact how effectively these guidelines are implemented to VTP. Despite global progress in reducing vertical transmission of HIV, inconsistent implementation of ART guidelines persists, particularly in maternity wards within private hospital settings (Kallon 2022). According to Mody et al. (2024), midwives play a crucial role in VTP, yet research has largely focused on public sector maternity services, leaving a gap in understanding how midwives working in maternity wards within private hospitals experience and implement the ART clinical guidelines and the guidelines for VTP of communicable infections 2023 (Aphane et al. 2025; Mody et al. 2024). This issue is especially relevant in sub-Saharan Africa, where the HIV burden remains high and healthcare delivery occurs across both public and private systems (Arora et al. 2021). Understanding these views is essential for strengthening vertical prevention and transmission programmes, as well as ensuring consistency. Thus, this study sought to explore the views of midwives working in maternity wards within private hospitals regarding the implementation of ART clinical guidelines to VTP. By understanding their perspectives, this study aims to identify potential barriers, facilitators and areas for improvement in the integration of these crucial HIV prevention strategies within private healthcare settings (Aphane et al. 2025; Mody et al. 2024). The study will also assess the level of awareness and training amongst healthcare providers regarding ART guidelines, as well as the role of institutional policies and resources in shaping their implementation, to provide ongoing efforts to eliminate vertical transmission. By understanding the gaps and challenges in the implementation process, this study seeks to provide valuable insights that could contribute to the development of strategies for improving the integration of ART guidelines in private maternity settings, enhancing training, policy development and the overall effectiveness of VTP programmes in private healthcare environments, ultimately improving patient outcomes and quality care.

## Research methods and design

### Research design

Qualitative research (Lim 2024), with an exploratory and descriptive design, was employed for the study. This approach was appropriate for understanding how midwives interpret and make sense of their experiences related to the implementation of ART clinical guidelines to VTP of HIV in maternity wards within a private hospital in Gauteng (Polit & Beck 2021). Qualitative research enabled the researchers to explore participants’ views in-depth and capture their subjective experiences within a real-world context.

### Study setting

This study was conducted in a private hospital located in Lynnwood Ridge, a suburb in the eastern part of Pretoria, within the Tshwane District of Gauteng province, South Africa. The City of Tshwane has a population of approximately 2.9 million residents and an estimated population density of about 1100 individuals per square kilometre (Fraser 2023). The hospital has 360 beds, including units such as accident and emergency, intensive care, neonatal intensive care, medical, surgical and maternity services. The maternity ward handles approximately 130 deliveries per month. The labour ward has seven antenatal beds and four delivery beds, whilst the postnatal ward has 33 beds. The Neonatal Intensive Care Unit (NICU) has 14 beds. Three departments, labour, postnatal and neonatal, employ a total of *n* = 37 midwives. However, midwives working in the neonatal ICU were excluded from the study due to differences in their roles. The labour and postnatal wards together had *n* = 20 midwives and *n* = 10 obstetricians who did not form part of the study.

### Study population and sampling strategy

The study population consisted of *n* = 13 registered midwives. Purposive sampling was employed to select participants based on their potential to provide rich, relevant information regarding the research objectives. Purposive sampling is a non-probability technique where participants are selected based on the researcher’s judgement about who can provide the most meaningful data (eds. Brink & Van Rensburg 2022). Midwives were selected because of their direct involvement in implementing ART guidelines in the maternity ward. The inclusion criteria included:

- All permanently employed midwives, including managers, registered with the South African Nursing Council (1990).
- Midwives and managers who had worked in the maternity wards within the private hospital for a minimum of 6 months.

The exclusion criteria included ancillary and auxiliary nurses, managers who do not work in the maternity wards, professional nurses, locum or temporary staff not working in maternity wards within the private hospital and midwives who did not consent to working in the study.

### Data collection

The researcher, Tshegofatso E. Chauke, visited the research site to recruit participants, who were invited to volunteer. A 2-day briefing session ensured that all potential participants were informed about the study’s purpose and ethical considerations. Data collection occurred between December 2024 and February 2025. All participants signed informed consent forms. Confidentiality, anonymity and the right to withdraw from the study at any time were guaranteed. Semistructured face-to-face interviews were conducted in a private room provided by the hospital to ensure confidentiality. With participants’ consent, interviews were audio-recorded and guided by a semistructured interview questionnaire. Each interview lasted approximately 20 min – 40 min, and scheduling was flexible to accommodate participants’ availability and service demands. Data saturation was reached after the 11th interview; however, two additional interviews were conducted to confirm that no new information was emerging.

### Data analysis

Data were analysed using Tesch’s method of qualitative data analysis, a systematic approach for organising and interpreting data derived from interviews and open-ended responses (Phehla, Makhene & Matshaka 2024). Data analysis commenced concurrently with data collection, allowing for the continuous identification and refinement of meaningful units of data. The researcher immersed herself in the data by repeatedly reading the transcripts to gain a comprehensive understanding of the content. Meaningful units were systematically coded and grouped into categories, from which themes and sub-themes emerged. Upon completion of the initial analysis, the transcripts and field notes were analysed by an independent coder. A consensus discussion was subsequently held between the researcher and the independent coder, during which agreement was reached on the final themes and sub-themes. Direct quotations from participants, presented in italics, were used to support the findings and to reflect the diverse perspectives of the participants.

### Ethical considerations

Ethical clearance for academic approval was obtained from the University of Pretoria Faculty of Health Sciences Research Ethics Committee (Ref: 204/2024) and approval for conducting research in the private healthcare was obtained by the Private Healthcare Research Committee (LHCHREC-PR-28052024/16). The selected hospital granted permission for the study to be conducted. Participants were fully informed of the study’s purpose and procedures. Participation was voluntary, with the option to withdraw at any time without penalty. To ensure anonymity, participants were not identified by name during the interviews. Instead, they were assigned unique identifiers that included participant number, gender, age, and professional role (e.g., P1, female, 54 years, midwife). No personal identifying information such as names was recorded. All interview data were stored securely to maintain confidentiality. Ethical principles of confidentiality, beneficence and non-maleficence were strictly upheld throughout the study.

## Results

### Participants’ characteristics

In this study, a total of *n* = 13 registered midwives participated in this study through semistructured, face-to-face interviews. The demographic data collected included age, gender, educational attainment, professional designation and years of experience working in maternity wards within the private hospital. Participants’ ages ranged from 25 to 55 years, and all were female. They were employed in both the labour and postnatal wards of the selected private hospital. Amongst the participants were an operational manager, an advanced midwife and 11 registered midwives. Their years of experience ranged from 6 months to 20 years, and their qualifications ranged from a 3-year basic diploma to a master’s degree in nursing. Table 1 summarises the demographic characteristics of the participants.

**TABLE 1:** Demographic characteristics of South African midwives who participated in the study (*N* = 13).

Variables	*n*	%
**Age group (years)**		
25–35	5	38.5
36–45	3	23.1
46–55	5	38.5
**Gender**		
Female	13	100.0
Male	0	0.0
**Educational attainment**		
3-year diploma	2	15.4
4-year diploma	3	23.1
Bachelor’s degree	8	61.5
**Professional designation**		
Operational manager	1	7.7
Advance midwife	1	7.7
Registered midwife	11	84.6
**Work experience in maternity ward**		
< 5 years	7	53.8
6–10 years	3	23.1
11–20 years	3	23.1

### Measures of trustworthiness

To ensure the trustworthiness of the study, the researcher applied Lincoln and Guba’s criteria (Enworo 2023), ensuring credibility through prolonged engagement, member checking and independent coding. Preliminary themes were shared with selected participants after initial analysis to verify whether interpretations reflected their experiences and views. Participant feedback was used to refine the themes, and an independent coder reviewed the findings to minimise researcher bias. Transferability: Ensured by providing detailed descriptions of the research context, participants and methodology. Dependability: Documented research processes and decisions to allow for replication. Confirmability: Maintained by incorporating direct quotes and using an audit trail to ensure that findings are grounded in the data.

### Findings of the study

Thematic analysis of the interview data yielded four key themes, each with associated sub-themes, reflecting the midwives’ experiences and perspectives on the implementation of ART clinical guidelines for VTP of HIV in private hospital settings. These themes highlight systemic gaps, ethical dilemmas, role limitations and practical strategies for improving HIV prevention practices. The themes are described in detail below, supported by direct quotations from participants to reflect their lived experiences and insights. These themes, along with their associated sub-themes, are summarised in Table 2 and discussed in detail in the subsequent sections.

**TABLE 2:** Outline of themes and sub-themes.

Themes	Sub-themes
**Theme 1: Midwives’ views on lack of standardised practice in implementing VTP guidelines**	1.1 Inconsistent adherence to established HIV management guidelines and policies 1.2 Disparities between private and public healthcare approaches to HIV testing 1.3 Irregular and inconsistent testing practices 1.4 Pre- and post-test counselling tasks shifted to laboratory technicians
**Theme 2: Ethical and confidentiality challenges**	2.1 Confidentiality versus patient non-disclosure 2.2 Persistent stigma and its impact on VTP HIV management
**Theme 3: Midwives’ views on their limited role in VTP management**	3.1 Restricted autonomy of midwives 3.2 Dependence on physicians’ orders and approval
**Theme 4: Strategies to enhance implementation/use of the ART clinical guidelines at private hospitals**	4.1 Training and awareness 4.2 Need for alignment of private hospital protocols with national VTP policies

ART, antiretroviral therapy; VTP, vertical transmission prevention.

#### Theme 1: Midwives’ views on the lack of standardised practice in implementing vertical transmission prevention guidelines

According to the study findings, the private hospital under investigation does not adhere to (do not use) any set protocols for VTP of HIV from mother-to-child during pregnancy, delivery, postpartum care or the breastfeeding phase. This lack of uniformity affects the quality and consistency of care. The following four sub-themes emerged from the data: (1.1) Inconsistent adherence to established HIV management guidelines and policies, (1.2) Disparities between private and public healthcare approaches to HIV testing, (1.3) Irregular and inconsistent testing practices, and (1.4) the shifting of pre- and post-test counselling to laboratory personnel.

**Sub-theme 1.1: Inconsistent adherence to established HIV management guidelines and policies:** Participants highlighted the absence of institutionalised protocols for HIV management in private maternity settings, noting that guidelines such as the 2023 ART Clinical Guidelines and the VTP guidelines were either unknown or not implemented. One respondent stated:

‘To be honest, I do not even know the guidelines. We do not have it. We don’t practice it. It’s not there. And I don’t even know the guidelines. I’ve never even seen the guideline …’ (P1, female, 54 years, midwife)

Another midwife reinforced this sentiment, drawing a contrast between the structured nature of public sector services and the fragmented approach observed in private facilities:

‘In government, there are protocols and ART clinical guidelines 2023 or Vertical Transmission Prevention guidelines 2023 that are followed. Now, they say that these guidelines are not in place. Remember that the patient is the physician’s patient here.’ (P2, female, 49 years, midwife)

**Sub-theme 1.2: Disparities between private and public healthcare approaches to HIV testing:** Midwives described significant differences in HIV testing protocols between the public and private sectors. In public hospitals, national ART and VTP guidelines are systematically implemented, and midwives are empowered to initiate testing and treatment. However, in the private sector, unclear responsibilities and fragmented systems often hinder such practices:

‘We don’t do the testing like in government. You know, every six weeks, you’ll do the tests. Here, we don’t do that … It’s not like in government where you know this is how you’re going to treat a patient based on their viral load, especially to prevent the risk of infection to the baby.’ (P4, female, 32 years, midwife)

‘Remember, in private, we are getting patients via the physicians. It’s unlike maybe in government, they were going to get first to the midwives, then you’re going to test them, and then you’re going to know their status.’ (P7, female, 53 years, midwife)

Another participant with experience in both the private and government sectors emphasised that there is a difference in the implementation of the guidelines:

‘It’s because I have both the private and public experience. I’d say that in the private sector, those who are least managed are … I’d say it’s not even adhered to, because you find, for example, that a mother comes in, and then you’re first and foremost concerned with these confidentiality clauses. In my opinion, it’s poorly managed in the private sector … They claim to do so, but you’d never see them. For example, in the government sector, there will be posters and guidelines showing you how to follow VTP. Maternal guidelines are intended for both private and public sectors, or at least that is what we are told. So why aren’t antenatal care [*ANC*] clinics in the private sector aligned with general ANC clinics?’ (P13, female, 35 years, midwife)

The ART clinical guidelines with integration of the guidelines for VTP of communicable infections may ensure that all pregnant women are routinely offered HIV testing and are linked to care promptly if needed. Moreover, public sector staff are often trained through nationally coordinated programmes, ensuring consistent practices across facilities. In contrast, midwives in private hospitals described HIV testing as being more influenced by patient preferences, physician discretion and institutional policies rather than standard national guidelines. In many cases, HIV testing is offered only upon request or after obtaining specific consent rather than as a routine part of antenatal care (Mshweshwe-Pakela et al. 2022). This difference not only delays testing and potential treatment initiation, but it may lead to missed opportunities for early diagnosis, especially in cases where patients are unaware of the importance of early testing.

**Sub-theme 1.3: Irregular and inconsistent testing practices:** The National Department of Health (2024), the National Integrated Maternal and Perinatal Care Guidelines for South Africa and the National Department of Health (2023), guidelines for VTP of communicable infections serve as a guide for implementing the prevention and treatment of vertical transmission. In the absence of clear policies and accountability structures, testing in private hospitals is largely discretionary. Midwives reported not being involved in HIV testing and not knowing how or when physicians tested their patients. This lack of standardisation may contribute to missed diagnoses and delayed interventions:

‘We do not do testing; we do not do HIV testing … So, it is only from the physicians word.’ (P4, female, 32 years, midwife)

‘We do not test them, so if they are a known patient to one of our gynaecologists, they have their routine when to test in pregnancy and when to do a follow-up test for CD4 counts. Therefore, we do not specifically state that this patient needs to be tested; it needs to come from the physician’s side.’ (P6, female, 25 years, midwife)

Another participant described how physicians’ personal biases influenced testing decisions:

‘You never know what happens. I do not think many physicians even test. One physician said, “Her husband is a reverend, so she doesn’t need to be tested.”’ (P9, female, 29 years, midwife)

Participants noted that whilst HIV testing is generally standardised and routine in public hospitals as part of comprehensive antenatal care, private hospitals often adopt a more fragmented, patient-driven approach.

**Sub-theme 1.4: Pre- and post-test counselling task shifted to lab technicians:** It is a concern when there are no clear channels of who does counselling within the private hospital. Midwives expressed concern over the inappropriate delegation of critical counselling responsibilities, particularly pre- and post-HIV test counselling to laboratory technicians; a practice observed in private hospital settings. This task shifting was reported to occur due to institutional practices that do not clearly define the roles and responsibilities in HIV care. Whilst lab technicians play an important role in diagnostic services, midwives felt that their training did not adequately prepare them to deliver the emotional, psychological and educational support required during HIV counselling sessions. Counselling is a vital component of the VTP cascade, as it ensures patients understand their diagnosis and the importance of ART adherence, helps mitigate stigma, reduces anxiety, provides safe infant feeding options and promotes partner testing. However, participants reported that this responsibility is often delegated to laboratory staff, who may not be adequately trained for such sensitive interactions:

‘It will be the lab people talking to the patient. The lab people, they come, and they do counselling, and the results are only disclosed to the physician whenever they get them.’ (P7, female, 53 years, midwife)

‘We do it with the lab, yes. So, when it’s done here, we call the lab, and they come with the consent form because it is very confidential … [*T*]hen they do the counselling. We do not do that.’ (P8, female, 36 years, midwife)

The midwives emphasised that pre- and post-test counselling should remain a clinical responsibility carried out by trained nurses or midwives, as they are better equipped to handle the sensitivity of HIV disclosure, psychosocial support and patient education. Delegating such roles may undermine trust and reduce the quality of care, particularly in a highly stigmatised context such as HIV.

#### Theme 2: Ethical and confidentiality challenges

This theme explores the ethical complexities experienced by midwives working in private maternity hospitals regarding HIV care and VTP. Whilst private hospitals aim to adhere to clinical and legal standards, including national VTP guidelines and confidentiality laws, the tension between patient rights and public health responsibilities often presents unique challenges. Two sub-themes emerged: (2.1) Confidentiality versus patient non-disclosure and (2.2) Persistent stigma and its impact on VTP HIV management.

**Sub-theme 2.1: Confidentiality versus patient non-disclosure:** Midwives in this study highlighted a complex ethical and legal dilemma they often face in the implementation of ART clinical guidelines for the VTP of HIV: the tension between maintaining patient confidentiality and managing the risks associated with non-disclosure of HIV status. This conflict is further intensified in private hospital settings, where adherence to strict confidentiality laws, such as the *Protection of Personal Information Act* (POPIA), is strictly enforced.

Whilst midwives are legally and ethically obligated to protect a patient’s personal and health information, they expressed concern about how this obligation sometimes limits their ability to prevent further HIV transmission, particularly when patients refuse to disclose their status to partners or family members.

‘We keep the patient’s status confidential, as the POPI Act guides us, but sometimes the husband is actively involved in her care and does not know she is HIV-positive. It is challenging knowing we cannot disclose, even when it puts others at risk.’ (P1, female, 54 years, midwife)

In these cases, midwives find themselves in emotionally and professionally challenging positions. Although they provide ongoing counselling and encourage voluntary disclosure, many patients fear social stigma, domestic violence or abandonment:

‘The POPI Act ties our hands, we respect the patient’s rights, but when she does not tell her partner, and he is part of the decision-making, it affects our ability to manage care holistically.’ (P2, female, 49 years, midwife)

However, some participants acknowledged that obstetricians occasionally disclose the patient’s condition, which makes their work easier, as they would know how to handle the newborn subsequently:

‘When we are aware as midwives of the patient’s HIV status, we act on it, and everything is carried out according to the national guidelines …’ (P3, female, 26 years, midwife)

**Sub-theme 2.2: Persistent stigma and its impact on vertical transmission prevention of HIV management:** Failure of a pregnant woman to disclose their HIV status to their partners, family members or friends due to fear of stigma or discrimination can hinder the effectiveness of the vertical transmission programme. Lack of partner involvement also reduces the effectiveness of the vertical prevention of HIV since an uninformed partner about their HIV status delays treatment and increases the risk of HIV transmission. HIV–related stigma remains a significant barrier to effective VTP. Participants in the study highlighted that patients’ fears of being judged, discriminated against or abandoned often lead them to withhold their HIV status even from their partners or the healthcare team. This secrecy severely compromises the timely implementation of VTP interventions. The following quotations are in support:

‘Well, sometimes when it’s something private like this, usually the HIV is somewhat stigmatised. Sometimes you tell them to, okay, since it’s this one you can keep, then you can take it at your regular time … HIV is still heavily stigmatised in our society. So, a patient still hides if they are taking treatment.’ (P3, female, 26 years, midwife)

Another midwife added:

‘I understand why they don’t want to disclose because there’s this whole stereotype and stigma regarding the status of HIV.’ (P6, female, 25 years, midwife)

Midwives expressed empathy for patients who choose non-disclosure due to fear of rejection or abuse. However, they also recognised that this lack of openness delays intervention, prevents early ART initiation for both mother and infant and limits the involvement of partners in care decisions, a crucial component of comprehensive VTP programmes.

Non-disclosure due to stigma can reduce adherence to ART, delay infant prophylaxis and increase the risk of vertical transmission. This sub-theme reinforces the need for both community-level stigma reduction campaigns and private sector protocols that support safe and supported disclosure, including partner counselling and involvement strategies (World Health Organization 2021). This sub-theme reinforces the need for both community-level stigma reduction campaigns and private sector protocols that support safe, supported disclosure, including partner counselling and involvement strategies.

#### Theme 3: Midwives’ views on their limited role in vertical transmission prevention management

Although the South African Nursing Council (2001) clearly defines midwives’ scope to include monitoring interventions to prevent infection transmission, participants reported that their independent role in managing maternal HIV care is greatly diminished in the private sector. Midwives expressed frustration over their passive roles, often limited to following obstetricians’ orders without actively participating in care decisions. Two key sub-themes emerged: (3.1) Limited midwife autonomy and (3.2) Reliance on physicians’ approval.

**Sub-theme 3.1: Restricted autonomy of midwives:** Midwives in private hospital maternity wards face considerable restrictions on autonomy, especially when caring for HIV-positive women during antenatal, intrapartum and postnatal care. Despite the existence of evidence-based, collaborative guidelines, the physician-driven approach often overrides midwives’ judgement and expertise. Midwives are frequently treated as assistants rather than independent practitioners, limiting their input in care planning. Hierarchies and institutional norms discourage midwives from making independent decisions due to legal and policy constraints. Additionally, the absence of midwife-led care models in private hospitals further sidelines their role, affecting care quality and continuity:

‘I suppose … [*T*]he dynamics in private hospitals are not the same as compared to the government institutions. Midwives here do not function independently. So, we don’t have like midwives’ patients … [*I*]t is a relationship between them and the physician.’ (P1, female, 54 years, midwife)

Several participants shared that their training in HIV and maternal care is underused, leading to reduced care quality, frustration, and low morale:

‘There are many circumstances where they’re just like: let me make it clear, I am the physician, you’re the nurse. We are capable of starting ART, but hospital policy doesn’t allow it without a physician’s order. Even in emergencies, we have to wait.’ (P8, female, 36 years, midwife)

‘We are trained to manage these cases, but our hands are tied. It feels like our role is limited to following instructions rather than making decisions. Should I decide on a patient’s care? Oh, that will not go down very well with the physician.’ (P9, female, 29 years, midwife)

Midwives questioned whether their autonomy could be restored, especially given their consistent presence throughout the maternity journey. Unlike the public sector, where they are empowered to initiate ART and provide counselling, the private sector remains physician-centric, contributing to fragmented HIV care and missed interventions.

**Sub-theme 3.2: Dependency on physicians’ orders and approval:** Midwives shared barriers to the independent implementation of ART clinical guidelines for the VTP in private hospitals as the over-reliance on physicians’ orders and approval. Despite being trained and competent in managing aspects of HIV care, including initiating ART and providing follow-up care, midwives often felt constrained by hospital hierarchies that required them to wait for a physician’s instruction before acting.

This dependency was particularly evident in cases where a newly diagnosed pregnant woman needed to be started on ART urgently. However, delays occurred due to the unavailability of a prescribing physician or unclear delegation protocols:

‘Absolutely, yes … [*W*]e are often expected to follow orders. The idea is to collaborate with physicians within the private hospital; my skills and knowledge should bring value, but that does not always happen.’ (P8, female, 36 years, midwife)

‘You wait for an order from the physician, then we carry out his orders. But as a midwife, we do not have that independent role to offer clinical suggestions to physicians, even in situations where their input could benefit the health and well-being of both the mother and the baby. You do not do that, instead you wait for the physicians’ orders.’ (P13, female, 35 years, midwife)

Interestingly, one midwife noted that not having autonomy in disclosing test results was somewhat of a relief, given the emotional difficulty of informing women about a positive HIV diagnosis:

‘I must tell you I do not want to be, the one telling the patient she tested positive for HIV … [*W*]hat if they have a mental breakdown?’ (P10, female, 40 years, midwife)

Midwives in this study advocated for greater clinical autonomy and role recognition, calling on private hospitals to re-evaluate their policies and shift toward a more inclusive and trust-based model of care.

#### Theme 4: Strategies to enhance the implementation or use of the antiretroviral therapy clinical guidelines at private hospitals

A key theme that emerged from the data was the urgent need for strategic improvements in how ART clinical guidelines, particularly those targeting the prevention of vertical HIV transmission (VTP), are implemented within private healthcare settings. Midwives emphasised that the lack of structured training, limited awareness and misalignment between institutional and national guidelines hindered their ability to provide consistent, evidence-based care. Two major sub-themes arose: (4.1) Training and awareness and (4.2) The need for alignment between private hospital protocols and national policies.

**Sub-theme 4.1: Training and awareness:** Midwives consistently identified training and awareness as a critical enabler or, when lacking, a barrier to the effective implementation of ART clinical guidelines for the VTP of HIV. In the private hospital context, many midwives reported limited opportunities for structured, updated training explicitly related to national HIV guidelines, including the latest guidelines on ART initiation, monitoring and infant feeding practices. Some participants indicated that whilst they received initial HIV-related training during their formal education, there had been few or no refresher courses, in-service workshops, or institutional updates provided by the private hospital. This lack of continuous professional development contributed to uncertainty, inconsistent practice and reduced confidence when managing HIV-positive pregnant women:

‘We don’t get regular updates or workshops like they do in public hospitals. So, sometimes we’re not sure if we are using the latest guideline or not.’ (P13, female, 35 years, midwife)

Others shared that awareness of national changes in VTP protocols often came informally, through colleagues or personal reading, rather than through official channels or institutional training sessions. This ad hoc approach to guideline dissemination increases the risk of outdated practices and inconsistent adherence across the facility:

‘I only found out about the new infant feeding recommendations through a friend at another clinic. There is no formal training here to update us.’ (P8, female, 36 years, midwife)

‘We last did that [*HIV training*] during our studies … but we don’t get any training whatsoever now regarding HIV prevention. I think we need in-service training and refresher courses, maybe involving physicians and management too.’ (P8, female, 36 years, midwife)

**Sub-theme 4.2: Need for alignment of private hospital protocols and national vertical transmission prevention policies:** Midwives working in private hospital settings often encounter discrepancies between national ART guidelines, particularly those concerning VTP of HIV, and the internal policies or guidelines of their health facilities. This misalignment can hinder the consistent and effective implementation of ART clinical guidelines, potentially compromising both maternal and child health outcomes. Participants reported ongoing challenges stemming from a lack of alignment between private hospital policies and national VTP guidelines, resulting in discrepancies in care protocols, delays in treatment and inconsistent HIV prevention practices.

‘Sometimes we know what the national guideline says, but our hospital still uses old protocols, and we do not know which one to follow.’ (P3, female, 26 years, midwife)

Midwives further explained that hospitals which do not formally adopt national guidelines also tend to exclude themselves from national training opportunities, resulting in fragmented professional development:

‘The government offers training for new ART guidelines, but our hospital doesn’t always participate because they say we have our system.’ (P8, female, 36 years, midwife)

Additionally, midwives cited the lack of systematic communication mechanisms for disseminating new or updated national policies to private facilities. Without institutional channels to receive and incorporate updated protocols, staff are left to rely on informal methods:

‘We only hear about new guidelines when someone brings a copy; there is no formal process to make sure we are updated.’ (P5, female, 55 years, midwife)

## Discussion

This study aimed to explore and describe the midwives’ views on implementing the antiretroviral clinical guidelines for VTP of HIV in a private hospital maternity ward, South Africa. According to the participants’ views, implementing the ART clinical guidelines within private hospitals was a great challenge, as they primarily follow orders from physicians. The study focused on addressing significant challenges in implementing the ART clinical guideline in VTP of HIV in maternity wards within the private hospital.

**Midwives’ views on the lack of standardised practice in implementing VTP guidelines** led to inconsistencies in care and potentially increased transmission rates from mother to child. The absence of institutional guidelines and the discretionary nature of HIV testing practices in private hospitals suggest that inconsistent systems may lead to delayed diagnosis, missed opportunities for intervention, and fragmented care delivery. The lack of a standardised approach undermines the overall effectiveness of VTP interventions and may contribute to preventable cases of mother-to-child transmission (Mbogua 2023). A study comparing quality improvement interventions between private and government hospitals highlighted significant differences in vertical transmission rates, emphasising the need for standardised protocols across all healthcare facilities (Iroz et al. 2024). The study findings suggest that private hospitals develop and implement standardised VTP guidelines tailored to their unique contexts, ensuring alignment with national VTP guidelines (National Department of Health 2023b) and international standards set by the World Health Organization (2021). Midwives emphasised the urgent need for the hospital to develop, adopt and enforce clear HIV management policies that align with national standards, accompanied by regular audits, staff training and supportive supervision (Mokone 2023). The counselling process is a cornerstone of VTP, as it helps address the psychological and social aspects of living with HIV during pregnancy (Cardenas et al. 2023). Furthermore, the delegation of counselling and testing responsibilities to non-clinical personnel may undermine patient trust and compromise the continuity of care, indicating the importance of clearly defined professional roles within VTP programmes. Regular education for the midwives on VTP best practices and perform regular audits to ensure adherence to established protocols should be conducted.

### Ethical and confidentiality challenges

Maintaining patient confidentiality and addressing ethical dilemmas are paramount in VTP (Balekang, Galvin & Rakgoasi 2025). Studies have shown that midwives play a crucial role in VTP, but their effectiveness can be compromised by ethical concerns and challenges in maintaining confidentiality (Matombo 2022). Midwives in private hospitals may not be fully aware of the latest ART guidelines or may have limited experience in managing HIV-positive pregnant women. The findings of this study show that midwives need ongoing training on ethical standards and confidentiality protocols related to HIV care to VTP (Aba Abraham & Clow 2022). There is a bigger issue than standardised guidelines only. Not all physicians who disclose the results were mentioned, and the midwives are not responsible for the treatment of the patients. With the current setting in the private sector, where the midwives are rather obstetric nurses and not autonomous midwives, this remains a physician issue. Midwives recommended that private hospitals develop clear institutional guidelines that balance POPIA compliance with ethical duties around safety, disclosure and partner involvement. They also called for more structured legal-ethical training and support systems to help navigate these dilemmas without compromising patient trust or the quality of care. Women may fear judgement from midwives or the community they come from, which could deter them from accessing comprehensive care. A supportive and non-judgemental environment is crucial to overcoming this challenge (Peiris & Gupta 2024). Offering supportive counselling services to address the emotional and ethical challenges faced by both patients and healthcare providers could be a solution to the arising problem (Nhemachena et al. 2023). To address these challenges, it is recommended that supportive counselling services be implemented, ethical training expanded, and non-judgemental, stigma-free environments fostered within maternity wards in private hospitals. Such efforts can empower midwives to deliver sensitive, ethically sound care whilst encouraging patients to engage more openly with treatment services.

### Midwives’ views on their limited role in vertical transmission prevention implementation and management

Midwives’ autonomy refers to the ability of nurses to make independent decisions regarding patient care, based on their clinical judgement and expertise (Taleghani et al. 2023). Participants described physician-dominated care models in private hospitals that restrict midwives’ decision-making authority, often preventing them from initiating ART, ordering tests or disclosing results even when clinically appropriate. Research have demonstrated that nurse-led interventions, including midwives, can significantly improve ART adherence amongst people living with HIV (Huang et al. 2024). Furthermore, enhancing midwives’ autonomy has been linked to increased job satisfaction, motivation, and professional development (Kim et al. 2022). Empowered midwives are more likely to engage in critical thinking and adapt care plans to meet individual patient needs, resulting in improved patient outcomes (Gittings et al. 2024). The perceived underutilisation of midwives’ skills may negatively affect morale, continuity of care and timely clinical interventions, suggesting that more collaborative care models could improve both staff engagement and patient outcomes. These findings reinforce the importance of aligning private sector practice with the professional scope defined by the South African Nursing Council (2001). A study by Mumuni Atoko et al. (2024) revealed that whilst midwives had positive perceptions and attitudes towards VTP services, there were concerns about their limited involvement in certain aspects of HIV care. According to Opoku-Danso and Habedi (2023), the findings support expanding midwives’ responsibilities in VTP care, including training in ART initiation and management to enhance their empowerment. White Vangompel et al. (2024) state that multidisciplinary collaboration encourages a team-based approach where midwives, obstetricians, paediatricians and counsellors work closely together in VTP efforts to improve birth outcomes.

### Strategies to enhance implementation and use of antiretroviral therapy clinical guidelines in private hospitals

Studies have shown that quality improvement interventions can significantly impact VTP services in both private and public hospitals (Morris et al. 2024). Participants indicated that lack of structured training, inconsistent dissemination of updated guidelines and poor integration with national programmes contribute to uncertainty and variation in practice. This disconnect not only undermines national HIV prevention goals but also reinforces inequities in maternal care between the public and private sectors. Aligning private hospital policies with national VTP guidelines would ensure consistency in care, reduce preventable transmissions and allow for a more unified health system response to HIV. The study’s findings encourage maternal education of HIV-positive pregnant women about the importance of ART in VTP. The education process should not only focus on the medical aspects, but also provide emotional support and address any fears or concerns regarding ART, side effects and the involvement of the partner (De Los Rios et al. 2021). In addition to clinical training, DiClemente-Bosco et al. (2022) said it is essential to train the midwives on practical communication skills to ensure that women feel comfortable discussing their HIV status and adhering to ART treatments. This training should include counselling techniques and strategies for reducing stigma and discrimination. The participants in the study further expressed that there should be links between public health programmes and private hospitals. Midwives suggested closer collaboration between private hospitals and the Department of Health, including invitations to national workshops, webinars and mentorship opportunities (Ferguson & Davidson 2023). This collaboration can ensure that patients have access to public health resources, including specialised HIV clinics, community outreach programmes and funding for ART medication, and women should wish to return to the public sector to continue with care (Obeagu & Obeagu 2024). Community Engagement Public health campaigns aimed at raising awareness of HIV transmission risks and ART’s role in prevention can help reduce stigma and increase demand for HIV testing and ART. Sensitisation of the community to reduce stigma and encourage HIV testing amongst pregnant women, supporting women in making informed reproductive choices, contributes to an enabling environment for effective HIV prevention. According to Aphane et al. (2025), Continuous Professional Development ensures that healthcare providers receive ongoing education on the latest ART guidelines and VTP strategies to enhance the quality of care they provide. Regular workshops, seminars and refresher courses for all healthcare providers ensure that they remain updated on the latest guidelines and best practices in preventing perinatal HIV transmission (Okusanya et al. 2022). Monitoring and evaluation are essential for assessing the effectiveness of training programmes, as they enable the evaluation of knowledge uptake and the identification of areas requiring improvement (Kuchenmüller et al. 2022). Regular evaluations can inform necessary adjustments to training content and delivery methods. In addition, there is a need to establish formal systems for the dissemination of updated ART and VTP guidelines from the Department of Health to private hospitals. Private hospitals should also be encouraged to participate in national VTP programmes, including training workshops, ART supply chains, and data reporting systems. Furthermore, private hospital protocols should be harmonised with national guidelines through periodic policy reviews and joint consultative forums. Establishing feedback mechanisms would allow midwives to report implementation challenges and receive guidance aligned with both institutional and national expectations (Nyante et al. 2024). Overall, a comprehensive approach that integrates policy alignment, continuous training, public–private collaboration, and community sensitisation is critical to enhancing the implementation and uptake of ART clinical guidelines in private healthcare settings (Clarke et al. 2023).

### Strengths and limitations

A key strength of this study was its in-depth exploration of midwives’ views on the implementation of ART clinical guidelines in the maternity wards within private hospitals, a relatively under-researched area in South Africa. The qualitative approach enabled a nuanced understanding of real-world challenges and provided valuable insights from frontline healthcare providers. However, the study was limited in scope, as it was conducted in one region of South Africa, and the findings may not be generalisable to other private hospitals or regions. In addition, the sample consisted solely of female participants, with no accoucheurs included, which may limit the diversity of perspectives. Furthermore, as with many qualitative studies, researcher bias may have influenced data collection or interpretation, despite efforts to maintain objectivity. Nonetheless, the study contributed valuable insights that may inform policy, practice and future research.

### Recommendations

To improve the services in private hospitals concerning the implementation of the ART Clinical guidelines to VTP of HIV, recommendations are made to support and improve clinical practice:

- Adopt and adapt standardised clinical guidelines aligned with national VTP protocols to ensure consistency across all healthcare sectors.
- Private facilities should ensure that national maternal and HIV management guidelines issued by the National Department of Health are fully implemented in maternity wards within private settings.
- Continuous professional development programmes should be strengthened to ensure midwives remain updated on ART guidelines, counselling approaches, and communication skills.
- Promote early and routine HIV testing and counselling throughout the antenatal, intrapartum and postpartum periods. Facilitate early ART initiation and systematic monitoring of pregnant women and their infants.
- Ensure adequate resources, including ART medication, HIV testing kits, access to updated protocols, strengthened patient education, psychosocial support (including stigma reduction counselling) and guidance on safe disclosure.
- Private hospitals should also utilise midwives to their full authorised scope of practice, as regulated by the South African Nursing Council (2001), to enhance timely care, continuity of services, and improved maternal and neonatal outcomes.
- Encourage collaboration with public health authorities, including joint participation in national HIV prevention programmes.
- Provide mentorship and supportive supervision to midwives to build capacity and confidence in managing HIV-related maternal care.
- Conduct further research to explore broader organisational and policy-related barriers that may hinder the effective implementation of ART clinical guidelines in the private sector.

## Conclusion

The implementation of ART clinical guidelines to VTP of HIV in private hospitals is essential for improving maternal and child health outcomes. Private hospitals may significantly contribute to the worldwide effort to end the HIV and AIDS epidemic by implementing these guidelines, which would also improve mother and child health outcomes and reduce vertical HIV transmission. Whilst challenges such as resource constraints, stigma and training gaps exist, the strengths of private hospitals, including better access to resources and more personalised care, provide a solid foundation for effective intervention. Participants recommended that improvement can be achieved through a standardised approach that includes the adoption of standardised clinical protocols, comprehensive staff training, early HIV testing and counselling, timely ART initiation, and postpartum care. It is equally important to integrate HIV care with routine maternal health services, ensure infrastructure and resources are in place and actively engage patients through education and support systems. The success of these interventions may depend on collaboration with public health authorities, a strong emphasis on monitoring and evaluation and the establishment of a stigma-free atmosphere.
